# Screening Mosquito House Entry Points as a Potential Method for Integrated Control of Endophagic Filariasis, Arbovirus and Malaria Vectors

**DOI:** 10.1371/journal.pntd.0000773

**Published:** 2010-08-03

**Authors:** Sheila B. Ogoma, Dickson W. Lweitoijera, Hassan Ngonyani, Benjamin Furer, Tanya L. Russell, Wolfgang R. Mukabana, Gerry F. Killeen, Sarah J. Moore

**Affiliations:** 1 School of Biological Sciences, University of Nairobi, Nairobi, Kenya; 2 Ifakara Health Institute, Ifakara, Tanzania; 3 Infectious and Tropical Diseases, London School of Hygiene and Tropical Medicine, London, United Kingdom; 4 University of Dar es Salaam, Dar es Salaam, Tanzania; 5 Department of Clinical Veterinary Science, Universität Bern, Bern, Switzerland; 6 School of Biological and Biomedical Sciences, Durham University, Durham, United Kingdom; 7 Liverpool School of Tropical Medicine, Liverpool, United Kingdom; London School of Hygiene and Tropical Medicine, United Kingdom

## Abstract

**Background:**

Partial mosquito-proofing of houses with screens and ceilings has the potential to reduce indoor densities of malaria mosquitoes. We wish to measure whether it will also reduce indoor densities of vectors of neglected tropical diseases.

**Methodology:**

The main house entry points preferred by anopheline and culicine vectors were determined through controlled experiments using specially designed experimental huts and village houses in Lupiro village, southern Tanzania. The benefit of screening different entry points (eaves, windows and doors) using PVC-coated fibre glass netting material in terms of reduced indoor densities of mosquitoes was evaluated compared to the control.

**Findings:**

23,027 mosquitoes were caught with CDC light traps; 77.9% (17,929) were *Anopheles gambiae sensu lato*, of which 66.2% were *An. arabiensis* and 33.8% *An. gambiae sensu stricto*. The remainder comprised 0.2% (50) *An. funestus*, 10.2% (2359) *Culex* spp. and 11.6% (2664) *Mansonia* spp. Screening eaves reduced densities of *Anopheles gambiae s. l*. (Relative ratio (RR)  = 0.91; 95% CI = 0.84, 0.98; *P* = 0.01); *Mansonia africana* (RR = 0.43; 95% CI = 0.26, 0.76; *P*<0.001) and *Mansonia uniformis* (RR = 0.37; 95% CI = 0.25, 0.56; *P*<0.001) but not *Culex quinquefasciatus, Cx. univittatus* or *Cx. theileri*. Numbers of these species were reduced by screening windows and doors but this was not significant.

**Significance:**

This study confirms that across Africa, screening eaves protects households against important mosquito vectors of filariasis, Rift Valley Fever and O'Nyong nyong as well as malaria. While full house screening is required to exclude *Culex* species mosquitoes, screening of eaves alone or fitting ceilings has considerable potential for integrated control of other vectors of filariasis, arbovirus and malaria.

## Author Summary

Mosquito vectors that transmit filariasis and several arboviruses such as Rift Valley Fever, Chikungunya and O'Nyong nyong as well as malaria co-occur across tropical Africa. These diseases are co-endemic in most rural African countries where they are transmitted by the same mosquito vectors. The only control measure currently in widespread use is mass drug administration for filariasis. In this study, we used controlled experiments to evaluate the benefit of screening the main mosquito entry points into houses, namely, eaves, windows and doors. This study aims to illustrate the potential of screening specific house openings with the intention of preventing endophagic mosquitoes from entering houses and thus reducing contact between humans and vectors of neglected tropical diseases. This study confirms that while full house screening is effective for reducing indoor densities of *Culex* spp. mosquitoes, screening of eaves alone has a great potential for integrated control of neglected tropical diseases and malaria.

## Introduction

Houses are the main site for contact between humans and night biting mosquito vectors [1], [2]. The impact of improved housing on indoor malaria vector densities [3]–[6] and transmission [7] is well established. In Africa, the primary malaria vectors are nocturnal, endophilic and endophagic mosquitoes of the *Anopheles gambiae* species complex [8], [9]. These vectors prefer to enter houses via open eaves [2]. Therefore, houses with open eaves or those lacking ceilings have higher numbers of mosquitoes and a greater malaria burden compared to those with closed eaves or with ceilings [3], [4], [7], [10].

Regardless of evidence that improved housing provides protection from Anopheles malaria vectors, its potential to reduce indoor biting densities of other mosquito genera has received little attention, despite the fact that several of these species are known vectors of diseases which cause significant morbidity and mortality. These diseases include lymphatic filariasis, several arboviruses such as Chikungunya, O'Nyong nyong, Rift Valley Fever (RVF) and West Nile Virus (WNV) (Table 1).

*An. gambiae sensu stricto* and *An. arabiensis* are the most abundant malaria vectors in rural tropical African countries and are also the main vectors of filariasis [11] as well as O'Nyong nyong [12]. *Mansonia africana* and *Ma. uniformis* are vectors of RVF and filariasis, although the latter predominantly transmits Brugian filariasis in Asia. Integrated control of filariasis and malaria is feasible [13], [14] due to their co-occurrence in rural areas, where they are often co-endemic and transmitted by the same vectors [15]. Though the main control measure against filariasis is chemotherapy, achieved through mass drug administration, a more holistic approach which integrates other proven interventions may be feasible in many endemic areas [16].

**Table 1:** Mosquitoes naturally infected with arboviruses or Bancroftian filariasis in southern and eastern Africa.

Species	Disease carried	Country	Reference
*Anopheles gambiae s.l.*	O'Nyong nyong	UgandaKenyaMozambique	[12]
	Bancroftian filariasis	Tanzania	[11]
*Anopheles funestus*	O'Nyong nyong	UgandaKenyaMozambique	[12]
	Bancroftian filariasis	Tanzania	[11]
*Mansonia africana*	Rift Valley Fever	Kenya	[38]
		Uganda	[39], [40]
	Chikungunya	Uganda	[12]
*Culex pipiens quinquefasciatus*	West Nile Virus	Madagascar	[50]
	Chikungunya	Tanzania	[51]
	Bancroftian filariasis	Tanzania	[11]
*Culex univittatus* complex	Sindbis Virus	South Africa	[52], [53]
	West Nile Virus	South Africa	[52], [54]
		Madagascar	[50]
		Kenya	[50], [55]
*Culex theileri*	West Nile Virus	South Africa	[12], [50]
	Rift Valley Fever	South Africa	[56]
*Culex rubinotus*	Witswatersrand	UgandaMozambiqueSouth Africa	[12], [39], [57]

*Culex quinquefasciatus* is a vector of *Wuchereria bancrofti* causing lymphatic filariasis in Africa. It is the main vector in urban areas [17] but also contributes to rural transmission. *Cx quinquefasciatus* is also a vector of other arboviruses such as Chikungunya and West Nile Virus (Table 1). Several other *Culex* species transmit other arboviruses in East Africa; these are shown in Table 1.

Crucially, culicines are also the major cause of nuisance biting in rural and especially urban areas [18]. Several studies have shown that the community is sensitive to changes in biting nuisance related to changes in mosquito densities. Uptake of several control measures such as use of house screens [19] and mosquito coils [20] is dependent upon the desire to prevent mosquito bites in addition to preventing diseases. Similarly, use of insecticide treated nets (ITNs) is motivated by the desire to prevent nuisance bites [21], [22], as shown by reduction in the use of ITNs when mosquito densities are lower due to seasonal decline, [23], [24] even when mosquito numbers are sufficient for disease transmission to continue.

Unfortunately, efficacy of insecticide based interventions declines when resistance develops, as has already been seen in Tanzania [25], [26]. If people continue to be bitten by nuisance mosquitoes due to development of insecticide resistance, it undermines public acceptance of ITNs as an intervention [27], [28]. Therefore, there is need to develop supplementary tools for control of nuisance mosquitoes. Reduction in nuisance mosquitoes will increase users' confidence in the available mosquito control measures and therefore also encourage use of other measures.

The aim of the study was to evaluate preferential points of entry of different mosquito species into houses. This was determined by indoor densities of different species of mosquitoes when a specific entry point was screened, precisely, eaves, windows and doors compared to an unscreened control. Our overall goal was to evaluate the optimal method needed for house screening in order to provide integrated control of filariasis, arboviruses and malaria vectors.

## Methods

### Study site

The experimental hut study was carried out at Lupiro village (8.01°S and 36.63°E) located in Ulanga district, in the south eastern part of Tanzania. The village lies 300 meters above sea level on the flood plain of Kilombero River, approximately 26 km south of Ifakara town. The climate is hot and humid, experiencing annual rainfall ranging between 1200–1800 mm and annual mean temperature between 20–32°C. This climate and the clearance of a perennial swamp for rice farming creates ideal conditions for perennially abundant populations of both *An. gambiae s. s*. and *An. arabiensis* and many species of culicine mosquitoes [29]. Malaria transmission intensity in this village is exceptionally high, averaging between 474 and 851 infectious bites per person per year, despite mosquito net coverage which consistently exceeds 75% [30]. In addition, there have been several cases of RVF and filariasis (E. Mossdorf pers comm).

### Local houses

**Figure 1:**
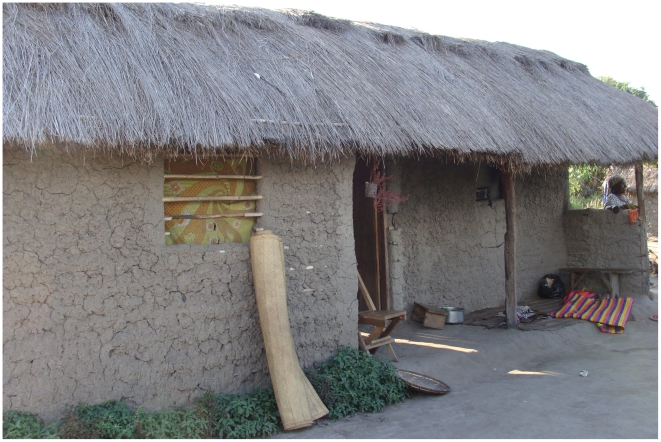
A local house. The local houses are made of mud walls and thatched roofs. They have one door and two windows and open eaves (open spaces between the roof and the wall).

In Ulanga and Kilombero DSS (Demographic Surveillance System) areas, most of the local houses have mud walls (56%), while the remainder are made of baked mud bricks. The roofs are mostly thatched (70%) or of corrugated iron. The houses chosen for these experiments therefore had mud walls and thatched roofs with open eaves and one or two windows (Figure 1). Cooking was mainly done outside of the hut and each of the local houses selected had two or three people living in them.

### Experimental huts

**Figure 2:**
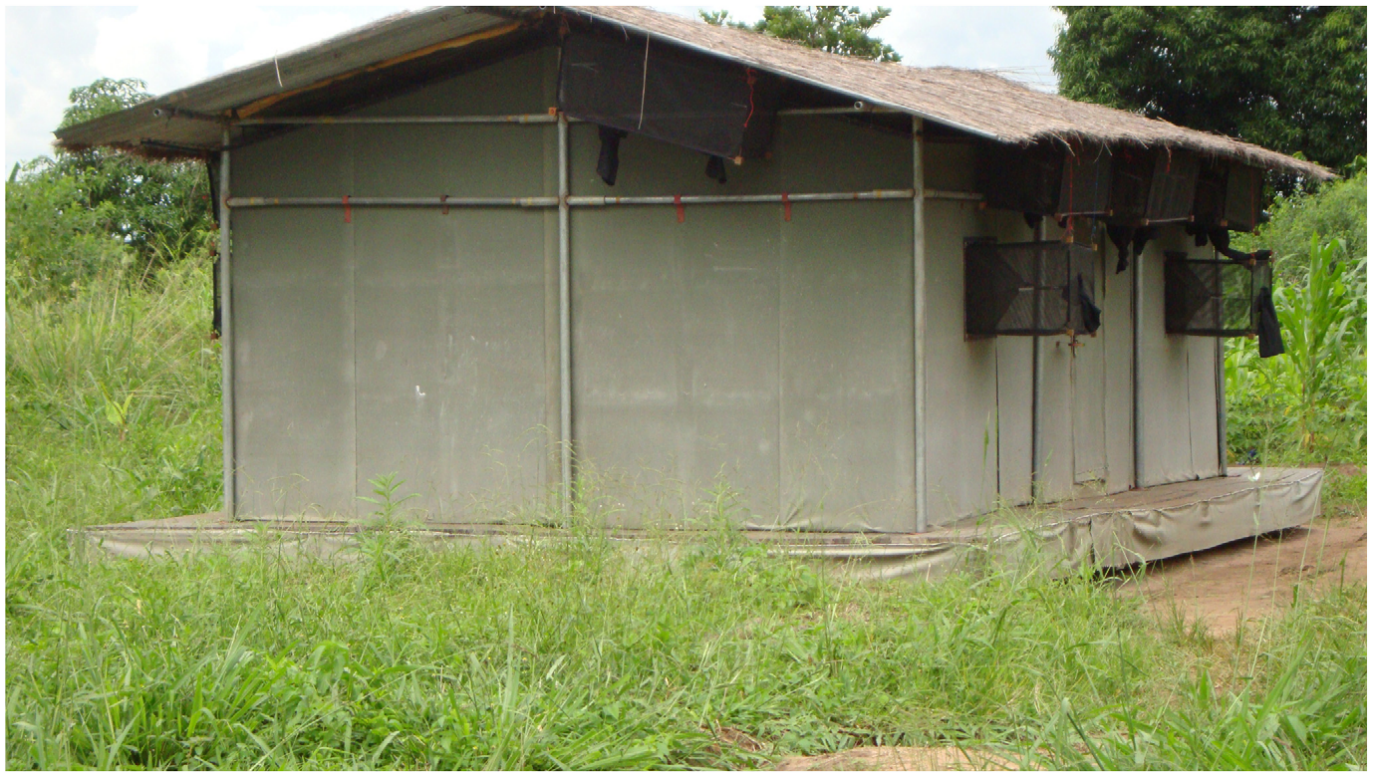
A wooden experimental hut. The experimental huts were designed to represent local housing in southern Tanzania An experimental hut had a corrugated roof and covered with grass thatch on the top, to simulate the temperature of local houses with thatched roofing. The outer walls were constructed from wooden planks or canvas. The inner walls were made of removable panels coated with mud. They had one door and two functional windows with open eaves (open spaces between the roof and the walls).

Several prototypes of a new design of experimental huts (Figure 2) (Moore *et al.*, Submitted) were built in Lupiro with the intention of representing, as closely as possible, the key structural features of local housing in southern Tanzania (i.e. brick or mud huts with corrugated iron or thatched roofing). These huts were designed in kit form for ease of portability, with a galvanized piping framework so that the entire hut could be flat packed. The roof is corrugated iron covered with grass thatch on the top, to simulate the temperature of local houses with thatched roofing. The outer walls are constructed from wooden planks or canvas. The inner walls are removable panels coated with mud, to simulate local mud walls. Two huts were constructed to mimic average local huts in the village. These were 6.5 m long, 3.5 m wide and 2 m high, (the size of these huts was determined by measuring 100 houses in Lupiro and calculating the average dimensions). The remaining two were smaller, at 3 m long, 3.5 m wide and 2 m high. The height of each structure measured 2.5 m at the roof apex. Each experimental hut had one door and two window openings as this was the median number seen in local houses.

### Experimental design

Two blocks of four huts were used for these experiments: one block of four local houses and one block of four experimental huts. The selected houses were located nearest to the experimental huts and were selected to be approximately 50 m apart from each other. Two male volunteers slept in each experimental hut. The volunteers were not rotated between huts but remained in the same hut for the duration of the study. The bias created by variation in human attractiveness to mosquitoes and spatial variation between huts were therefore combined and treated as a single source of bias in the statistical analysis. For each of the two blocks of four houses, the following sequence of experimental treatments was completed. In each block, four repetitions of four experimental treatment arrangements were completed between 4^th^ December and 19^th^ December 2007. This is the peak of short rains and therefore there is wide spread flooding leading to high densities of mosquitoes of all genera. Each repetition included three nights during which three of the four houses had the same one of the three potential entry points screened while the remaining fourth house was completely unscreened. On the first night of each repetition, all the four huts remained completely unscreened. For the subsequent three nights of each repetition, all the three treatments were changed each night from screening the eaves to windows and then doors, in that order. For each night, a different hut was chosen within each block to have no entry point screened, so that at the end of the four repetitions, all four huts had acted as these contemporaneous controls. The treatments were rotated across all the huts systematically. Rotation of treatments reduced the bias of mosquito collections between the huts.

### Screening entry points

PVC-coated fibreglass netting material (Elastic Manufacturing, Tanzania) was used to screen specific entry points each particular night. The netting was cut to fit each of the entry points (doors windows and eaves). In the experimental huts, the size of the windows, eaves and doors was uniform for all the huts. Screens were fitted on the experimental huts by hook and loop fasteners. In the local houses, the screens were nailed onto the wall (mud wall). The nails could be removed easily each morning at the end of the experiments. Due to uneven wall surfaces of the local huts, small gaps were found between the netting and the wall. These gaps were blocked with cotton wool to create a complete barrier.

### Mosquito collection

CDC light trap is an appropriate tool for sampling mosquito vectors that would otherwise bite humans, thus being comparable to human landing catches [31]–[34]. A CDC miniature light trap (model 512) was positioned approximately 1 m above the ground. It was placed next to the bed (at the foot end) occupied by an adult male volunteer, under an untreated bed net [32]. Volunteers operated light traps from 19:00 to 07:00 hrs each night.

Although no attempt was made to control times at which occupants slept, this period typically approximated 19:00 hrs to 07:00 hrs. Traps were collected from each house every morning at 07.00. Collection bags were then placed in a plastic bucket, and mosquitoes were killed using cotton wool treated with chloroform.

### Mosquito identification

The mosquitoes were morphologically identified to genus level each morning in the field while they were still fresh. Mosquitoes were stored in small centrifuge tubes which contained tissue paper with silica gel beneath, then transported to the laboratory where they were stored at −20°C, until further identification. Further identification was done to species level using polymerase chain reaction (PCR) for *An. gambiae s. l*. [35]. Mosquitoes allocated for PCR were sampled randomly from *An. gambiae s. l*., mosquitoes collected from different trap nights by placing labelled tubes in a box and picking them at random. Morphological identification of culicines was done using a key [36].

### Ethics

Volunteers were recruited only if they agreed to participate in the study and signed a written informed consent form. To minimize risk of infection of mosquito borne diseases, participants were provided with untreated nets. In addition, they were offered free malaria screening and treatment. Ethical approval was granted by Ifakara Health Institute (IHI) (IHRDC/IRB/No. A-014-2007, IHRDC/IRB/No.A-019-2007) and the National Institute of Medical Research (NIMR/HQ/R.8a/Vol. W710). Centre for Disease Control (CDC) ethical review deemed the work non-human subjects research.

### Statistical analysis

Generalized estimating equations were used with SPSS 15 to estimate the effect of screening specific entry points, which was treated as a categorical independent variable, on indoor mosquito densities relative to unscreened controls. House number was fitted as a subject effect and day as the within-subject variable, with an exchangeable working correlation matrix, to account for spatial and temporal heterogeneity in the dependent variable, namely number of mosquitoes of a given mosquito taxon caught in each house on each night. Note that, each species was analyzed separately using generalised estimating equation model. *An. gambiae s. l*. mosquito catch had a normal distribution and was fitted to an identity link. All the other species were negatively skewed and were therefore fitted with a negative binomial and a log link function. The model was used to derive the relative rates and their 95% confidence intervals.

Binary logistic regression was used to test the strength of the influence of different treatments on the proportion of *An. arabiensis* and *An. gambiae s. s* caught, that were identified to sibling species by PCR. The independent variables fitted in the model were treatment and house number. The outcome variable was binomial; *An. arabiensis* and *An. gambiae s. s* were coded as 1 and 0 respectively and the effect of treatment on the odds ratio of finding *An. arabiensis* relative to *An. gambiae s. s.* was calculated.

## Results

### Mosquito collections

During the cumulative 16 nights of sampling, with the CDC light traps, 77.9% (17,929) of the total catch were *Anopheles gambiae s. l.* This species complex comprised 66.2% (738) *An. arabiensis* and 33.8% (n = 377) *An. gambiae s. s* (n = 1115 successful PCR amplifications). There were only 0.2% (n = 50) *An. funestus* species complex caught in the entire study. One tenth (10.2%, n = 2359) of all mosquitoes collected were various *Culex* spp. Three quarters (76.9%) of *Culex* spp. were identified as *Cx. pipiens* complex of which four fifths (80.3%, n = 875) were *Cx pipiens quinquefasciatus* while the remainder (19.7%, n = 214) were *Cx. pipiens pipiens*. Other culicines included *Cx. univittatus* and *Cx. theileri* (20.0% of the total *Culex* spp). Just over one tenth (11.6%) of all mosquitoes collected were *Mansonia* spp., of which more than half (58.3% n = 1038) were *Ma. uniformis* and the remaining 41.6% (n = 742) were *Ma. africana*. Other species of culicines caught in smaller numbers were, *Cx. horridis* (n = 7), *Cx. andersanius* (n = 11), *Cx. acrostichalis* (n = 43), *Cx. rubinotus* (n = 30), *Cx. sitiens* (n = 5), *Cx. simpsoni* (n = 18), and *Cx. aureus* (n = 69).

### Effect of screening different entry points on indoor densities

A summary of the median indoor density species collections when each entry point was screened is presented in Table 2 and a statistical estimate of the impact of screening is presented in Table 3.

*An. gambiae s. l.* mosquitoes were less likely to be found in houses with screened eaves (Table 3). Binary logistic regression revealed that both treatment (screening of various entry points) and house did not affect the proportion of *An. gambiae s. s.* versus that of *An. arabiensis* mosquitoes, (Treatment, Odds Ratio [95% confidence interval]  = 1.06 [0.94, 1.20]; Wald Chi square = 0.87; *P* = 0.35), indicating that the effect of treatment on the two sibling species was similar. Screening eaves also reduced both *Ma. africana* and *Ma. uniformis* mosquito densities by almost half (Table 3). Screening windows and the door reduced indoor densities of *Cx. quinquefasciatus*, *Cx. theileri* and *Cx. univittatus* mosquito densities by a quarter or more although this was not significant (Table 3). The relative densities of *Cx. univittatus* and *Cx. theileri* mosquitoes were increased when eaves were screened respectively (Table 3).

**Table 2:** Median indoor densities of different mosquito species caught in experimental huts and local houses when different entry points were screened.

Screened entry point		None^a^		Eaves		Windows		Door
Hut nights (N)		56		24		23^b^		24
Mosquito species	N	Median[IQR]	n	Median[IQR]	n	Median[IQR]	n	Median[IQR]
*An. gambiae sensu lato.*	8341	80.0[4, 630]	2708	59.0[9, 415]	2946	80.0[15, 370]	3934	96.0[17, 700]
*Ma. Africana*	336	3.0[0, 31]	144	0.0[0, 12]	138	3.0[0, 24]	144	1.0[0, 36]
*Ma. Uniformis*	584	3.5[0, 66]	93	1.5[0, 21]	198	6.0[0, 36]	163	1.5[0, 37]
*Cx. quinquefasciatus sensu lato.*	544	2.0[0, 79]	206	2.0[0, 40]	171	2.0[0, 46]	168	0.0[0, 50]
*Cx. Theileri*	27	0.0[0, 5]	28	0.0[0, 8]	4	0.0[0, 2]	9	0.0[0, 5]
*Cx. Univittatus*	60	0.0[0, 11]	49	0.5[0, 10]	19	0.0[0, 5]	16	0.0[0, 4]

N =  Number of hut nights of experimentation conducted for each treatment.

n = number of mosquitoes caught.

a Reference group (No entry point was screened).

b A CDC Light trap was attacked by ants on one of the nights, thus no data was recorded for that particular hut night so N = 23 rather than 24.IQR  =  Interquartile range

**Table 3:** Impact of screening various entry points upon indoor densities of different mosquito species caught with reference to indoor densities when no entry point was screened.

Screened entry point	None^a^	Eaves		Windows		Door	
Mosquito species	RR	RR[95%CI]	P	RR[95%CI]	P	RR[95%CI]	P
*An. gambiae sensu lato*	1	**0.91[0.84, 0.98]**	**0.01**	0.98[0.94,1.02]	0.34	1.03[0.97, 1.09]	0.31
*Ma. Africana*	1	**0.43[0.26, 0.76]**	**<0.001**	0.91[0.58,1.44]	0.70	1.03[0.63, 1.70]	0.90
*Ma. Uniformis*	1	**0.37[0.25, 0.56]**	**<0.001**	0.85[0.54, 1.33]	0.47	0.65[0.38, 1.13]	0.13
*Cx. quinquefasciatus sensu lato*	1	0.91[0.50, 1.65]	0.74	0.77[0.42, 1.39]	0.38	0.72[0.36, 1.45]	0.36
*Cx. theileri*	1	**2.42[1.13, 5.18]**	**0.02**	0.36[0.11, 1.22]	0.10	0.78[0.25, 2.46]	0.67
*Cx. univittatus*	1	**1.91[1.05, 3.47]**	**0.04**	0.77[0.37, 1.61]	0.49	0.62[0.31, 1.25]	0.18

The relative rates (RR), model estimated 95% confidence intervals (CI) and probability of equivalence (P) were all estimated by Generalized estimating equations as described in the Methods section.

a =  Reference group (No entry point was screened).

## Discussion

More than three quarters of the mosquitoes caught during the study were *An. gambiae s. l.* a major vector of both lymphatic filariasis as well as malaria in this area and across most of Africa [11]. *An. funestus* complex mosquitoes caught in this study were not identified to species level. However, other studies from Tanzania have shown that this species complex shows distinct behavioural differences. *An. funestus s. s.* mosquitoes are mainly endophagic while others like *An. rivulorum* are mainly exophagic [37]. Therefore, since mosquitoes were collected indoors we assume that most of the mosquitoes caught were *An. funestus s. s.*

Culicine mosquitoes collected in this study contribute to the transmission of filariasis and arboviruses (Table 1). *Cx. quinquefasciatus* was the most abundant *Culex* species caught. Significant numbers of *Cx. univittatus* and *Cx theileri* mosquitoes were also caught. *Ma. africana* has been incriminated as a vector of RVF [38]–[40], and was present in high densities during an outbreak of RVF among humans at the field site (E. Mossdorf pers comm).

Most of the mosquitoes caught were unfed, and therefore considered to be caught in the act of host seeking [31], [34]. Studies carried out previously in the same experimental huts (unpublished data) indicated that there were very low densities of indoor resting mosquitoes. Only 0.35% of the mosquitoes caught in that particular study were caught resting. Therefore it may be assumed that indoor resting mosquitoes were present in insufficient numbers to bias the outcome of the screening experiments.

Consistent with previous reports [3]–[5], *Anopheles gambiae s. s*. and *An. arabiensis* mosquitoes were noted to prefer eaves as the main entry point, demonstrated by reduced indoor densities when this particular entry point was screened. Both *Ma. africana* and *Ma. uniformis* also preferred entry via eaves as exhibited by reduced indoor densities when eaves were screened. This data indicates that transmission of the diseases these vectors transmit could be prevented by blocking eaves [2].

A study carried out in the Gambia showed a reduction in culicine indoor densities in houses with closed eaves but in association with horses tethered outside and with increased room height [41]. Indoor *Cx. pipiens s. l.* densities were reduced by 38% when eaves were closed [41]. On the contrary, a second study recently carried out in The Gambia measured the impact of closing eaves in addition to screening the doors in houses with no windows. The same study indicated that there was no additional reduction in culicine mosquito densities when eaves were blocked [42]. In the present study, we have shown that *Cx. quinquefasciatus*, *Cx. univittatus* and *Cx. theileri* mainly prefer windows and doors as their main point of entry. It is also important to note that when eaves were screened and windows and doors were left open, indoor densities of *Cx. univittatus* and *Cx. theileri* mosquitoes were increased in comparison to when all the three entry points were left unscreened. This indicated the importance of screening all the three entry points to achieve control of *Culex* spp. mosquitoes.

Effectiveness of house proofing on mosquito vectors depends on the interaction between their feeding behaviour and human behaviour especially when and where people eat and sleep [43]–[45]. House screening will only reduce exposure to endophagic mosquito vectors. Several anophelines in Africa are endophagic; therefore, house screening would be highly effective. Since most *Culex* spp. mosquitoes are commonly thought to be predominantly exophagic, then it raises concerns of whether house screening would be effective against them. However, varying levels of both endophagy and exophagy observed in different species; differ from one region to another. In East and West Africa *Cx quinquefasciatus* is more endophagic [46]. *Cx. univittatus* and *Cx theileri* exhibit both exophagy and endophagy in some areas [47]–[49]. In addition, our study also demonstrates endophagy by these *Culex* species.

Our findings suggest that screening eaves reduces indoor densities of *Anopheles gambiae s. l*. as well as *Mansonia* spp. both of which are vectors of several neglected tropical diseases in rural areas of Africa and some parts of Asia. Blocking eaves and full house screening, as a control tool against mosquito vectors may reduce nuisance mosquitoes and thus encourage uptake of control interventions which rely on acceptance, participation and even investment by the community.

Screening of eaves and/or installation of ceilings may prove to be practical and affordable where existing house designs prove amenable to such modifications. While most of the African population does not live in houses as uniform as our experimental huts, it is encouraging that mosquito proofing of houses by screening the eaves or installing ceilings has proven equally effective for anophelines and some culicines in rural settings in both East and West Africa. Blocking the eaves of the mud-walled, thatch-roofed village houses included in this Tanzanian study yielded results which are remarkably consistent with those observed when netting ceilings and screened eaves were installed into typical houses in The Gambia despite the wide geographical separation between them [3].

Recent evidence from urban Dar es Salaam [19] suggests that communities perceive closed ceilings and window screening as successful means to prevent house entry by mosquitoes. They demonstrate high levels of acceptance, uptake and even investment, despite the fact that this intervention has never been specifically promoted on this basis. We suggest that the true full potential of protecting houses against house entry by culicine and anopheline mosquitoes, could be better achieved through insecticide treated screening material for targeted killing by placing them on either eaves, windows and doors.

## References

[1] Gamage-Mendis AC, Carter R, Mendis C, De Zoysa AP, Herath PR (1991). Clustering of malaria infections within an endemic population: risk of malaria associated with the type of housing construction.. Am J Trop Med Hyg.

[2] Snow WF (1987). Studies on the house-entering habits of mosquitoes in The Gambia, West Africa: experiments with prefabricated huts with varied wall apertures.. Med Vet Entomol.

[3] Kirby MJ, Green C, Milligan MP, Chalarombos S, Jasseh M (2008). Risk factors for house entry by malaria vectors in rural town and satellite villages in Gambia.. Malar J.

[4] Lindsay SW, Emerson PM, Charlwood JD (2002). Reducing malaria transmission by mosquito-proofing homes.. Trends Parasitol.

[5] Lindsay SW, Jawara M, Paine K, Pinder M, Walraven GE (2003). Changes in house design reduce exposure to malaria mosquitoes.. Trop Med Int Health.

[6] Ghebreyesus TA, Haile M, Witten KH, Getachew A, Yohannes M (2000). Household risk factors for malaria among children in the Ethiopian highlands.. Trans R Soc Trop Med Hyg.

[7] Kirby MJ, Ameh D, Bottomley C, Green C, Jawara M (2009). Effect of two different house screening interventions on exposure to malaria vectors and on anaemia in children in The Gambia: a randomised controlled trial.. The Lancet.

[8] Gillies MT, DeMeillon B (1968). The *Anophelinae* of Africa South of the Sahara (Ethiopian zoogeographical region)..

[9] Day JF (2005). Host seeking strategies of mosquito disease vectors.. J Am Mosq Control Assoc.

[10] Lindsay SW, Snow RW (1988). The trouble with eaves: house entry by vectors of malaria.. Trans R Soc of Trop Med Hyg.

[11] White GB (1971). Chromosomal evidence for natural interspecific hybridization by mosquitoes of the *Anopheles gambiae* complex.. Nature.

[12] CDC (2009). Arbovirus catalogue.

[13] Muturi EJ, Mbogo CM, Nganga Z, Kabiru E, Mwanadawiro C (2006). Relationship between malaria and filariasis transmission indices in an endemic area along the Kenyan coast.. J Vect Borne Dis.

[14] Muturi EJ, Jacob BG, Kim C, Mbogo C M, Novak JR (2008). Are co-infections of malaria and filariasis of any epidemiological significance?. Parasitol Res.

[15] White GB (1989). Lymphatic filariasis in Geographical Distribution of Arthropod-borne Diseases and their principal vectors..

[16] Kolstrup N, McMatton JE, Magayuka SA, Mosha WF, Bushrod FM (1981). Control measures against Bancroftian filariasis in Coastal villages in Tanzania.. Ann Trop Med Parasitol.

[17] Hamon J, Burnett GF, Adam JP, RickenBach A, Grjebine A (1967). *Culex pipiens fatigans* Wiedemann, *Wuchereria bancrofti* Cobbold, et le development economique de l'Afique tropicale.. Bull WHO.

[18] Chavasse DC, Lines JD, Ichimori K, Majala AR, Minjas JN (1995). Mosquito control in Dar es Salaam. II. Impact of expanded polystyrene beads and pyriproxyfen treatment of breeding sites on *Culex quinquefasciatus* densities.. Med Vet Entomol.

[19] Ogoma BS, Kannady K, Sikulu M, Chaki P, Govella NJ (2009). Window screening, ceilings and closed eaves as sustainable ways to control malaria in Dar es Salaam, Tanzania.. Malar J.

[20] Chavasse DC (1996). The relationship between mosquito density and mosquito coil sales in Dar es Salaam.. Trans Roy Soc Trop Med Hyg.

[21] Van Bortel W, Barutwanayo M, Delacollette C, Coosemans M (1996). Motivation to acquire and use impregnated mosquito nets in a stable malaria zone in Burundi.. Trop Med Int Health.

[22] Adongo PB, Kirkwood B, Kendall C (2005). How local community knowledge about malaria affects insecticide-treated net use in northern Ghana.. Trop Med Int Health.

[23] Frey C, Traore C, De Allegri M, Kouyate B, Mueller O (2006). Compliance of young children with ITN protection in rural Burkina Faso.. Malar J.

[24] Aikins MK, Pickering H, Alonso PL, D'Allesandro U, Lindsay SW (1993). A malaria control trial using insecticide-treated bed nets and targeted Chemoprophylaxis in a rural area of The Gambia.. Trans R Soc of Trop Med Hyg.

[25] Kulkami MA, Malima R, Mosha WF, Msangi S, Mrema E (2007). Efficacy of pyrethroid-treated nets against malaria vectors and nuisance-biting mosquitoes in Tanzania in areas with long-term insecticide-treated net use.. Trop Med Int Health.

[26] Mosha WF, Lyimo N, Oxborough R, Malima R, Tenu F (2008). Experimental hut evaluation of the pyrrole insecticide chlorfenapyr on bed nets for the control of *Anopheles arabiensis* and *Culex quinquefasciatus*.. Trop Med Int Health.

[27] Schellenberg JR, Abdulla S, Minja H, Nathan R, Mukasa O (1999). KINET: a social marketing programme of treated nets and net treatment for malaria control in Tanzania, with evaluation of child health and long-term survival.. Trans R Soc Trop Med Hyg.

[28] Myamba J, Maxwel CA, Asidi AN, Curtis CF (2002). Pyrethroid resistance in tropical bedbugs, *Cimex hemiptera*, associated with use of treated bednets.. Med Vet Entomol.

[29] Killeen GF, Smith TA, Ferguson HM, Mshinda H, Abdulla S (2007). Preventing childhood malaria in Africa by protecting adults from mosquitoes with insecticide-treated nets.. PLos Med.

[30] Killeen GF, Tami A, Kihonda J, Kasigudi N, Ngonyani H (2007). Cost-sharing strategies combining targeted public subsidies with private-sector delivery achieve high bednet coverage and reduced malaria transmission in Kilombero Valley, southern Tanzania.. BMC Infect Dis.

[31] Lines JD, Curtis CF, Wilkes TJ, Njunwa KJ (1991). Monitoring human-biting mosquitoes (Diptera: *Culicidae*) in Tanzania with light-traps hung beside mosquito nets.. Bull Entomol Res.

[32] Mboera LE, Kihonda J, Braks MA, Knols BG (1998). Short report: Influence of centres for disease control light trap position, relative to a human-baited bed net, on catches of *Anopheles gambiae* and *Culex quinquefasciatus* in Tanzania.. Am J Trop Med Hyg.

[33] Okumu OF, Kotas EM, Kihonda J, Mathenge E, Killeen GF (2008). Comparative Evaluation of Methods Used for Sampling Malaria Vectors in the Kilombero Valley, South Eastern Tanzania.. The open Tropical Medicine Journal.

[34] Davis JR, Hall T, Chee EM, Majala A, Minjas J (1995). Comparison of sampling Anopheline mosquitoes by light-trap and human-bait collections indoors at Bagamoyo, Tanzania.. Med Vet Entomol.

[35] Scott JA, Brogdon WG, Collins FH (1993). Identification of single specimens of the *Anopheles gambiae* complex by the polymerase chain reaction.. Am J Trop Med Hyg.

[36] Edwards FW (1941). Mosquitoes of the Ethiopian Region. Culicine adults and pupae.. London British Museum.

[37] Kamau L, Munyekenye GO, Koekemoer LL, Hunt RH, Coetzee M (2003). A survey of the *Anopheles funestus* (Diptera: *Culicidae*) group of mosquitoes from 10 sites in Kenya with special emphasis on population genetic structure based on chromosomal inversion karyotypes.. J Med Entomol.

[38] Logan TM, Linthicum KJ, Davies FG, Binepal YS, Roberts CR (1991). Isolation of Rift valley Fever Virus from Mosquitoes (Diptera: *Culicidae*) collected during an outbreak in domestic animals in Kenya.. J Med Entomol.

[39] Hernderson BE, McCrae AWR, Kirya BG, Ssenkubuge Y, Sempala SDK (1972). Arboviruses epizootics involving man mosquitoes and vertebrates at Lunyo, Uganda 1968.. Ann Trop Med Parasitol.

[40] Williams MC, Woodall JP, Cobert PS, Haddow AJ (1960). An outbreak of Rift Valley fever occurring near Entebbe: Entomological studies and further isolations.. East African Virus Research Institute Report.

[41] Kirby MJ, West P, Green C, Jasseh M, Lindsay SW (2008). Risk factors for house-entry by culicine mosquitoes in a rural town and satellite villages in the Gambia.. Parasit Vectors.

[42] Njie M, Dilger E, Lindsay SW, Kirby MJ (2009). Importance of Eaves to House Entry by Anopheline, But Not Culicine, Mosquitoes.. J Med Entomol.

[43] Govella NJ, Okumu FO, Killeen GF (2010). Insecticide-Treated Nets Can Reduce Malaria Transmission by Mosquitoes Which Feed Outdoors.. The American Journal of Tropical Medicine and Hygiene.

[44] Winney R (1960). The biological activity of mosquito coils based on pyrethrum and coils based other active ingredients.. Pyrethrum Post.

[45] Pates H, Curtis C (2005). Mosquito behaviour and vector control.. Annu Rev Entomol.

[46] Subra B (1981). Biology and control of *Culex pipiens quinquefasciatus* Say, 1823 (Diptera, *Culicidae*) with special references to Africa.. Insect Science Application.

[47] McIntosh BM, Jupp PG, Dickinson DB, McGillivray GM, Sweetnam J (1967). Ecological studies on sindbis and West Nile viruses in South Africa.1. Viral Activity as revealed by Infection of Mosquitoes and sentinel Fowls.. South African Journal of Medicine and Science.

[48] Gad MA, El Said S, Soliman B, N Hassan A, Shoukry A (1987). Distribution and bionomics of Egyptian *Culex univittatus* (Theobald).. Journal of the Egyptian Society of Parasitology.

[49] Rifaat MA, Mahdi AH, Wassif SF, Talib MI (1969). Certain studies on *Culex univittatus* in relation to filariasis in the Nile Delta, UAR.. Journal of Egypt public health Association.

[50] Burt FJ, Grobbelaar AA, Leman PA, Anthony FS, Gibson GVF (2002). Phylogenetic relationship of Southern African West Nile Virus isolates.. Emerg Infect Dis.

[51] Ross RW (1956). The Newala epidemic. 111. The virus isolates, pathogenic properties and relationship to the epidemic.. J Hyg (Lond).

[52] Jupp PG, Blackburn NK, Thompson DL, Meenehan GM (1986). Sindbis and West Nile Virus Infections in the Witwatersrand-Pretoria Region.. South African Medical Journal.

[53] McIntosh BM, Jupp PG, De Sousa J (1972). Further isolations of arboviruses from mosquitoes collected in Tongaland, South Africa, 1960-1968.. J Med Entomol.

[54] Jupp PG, McIntosh BM, Blackburn NK (1986). Experimental assessment of the vector competence of *Culex* (Culex) *neavei* Theobald with West Nile and Sindbis viruses in South Africa.. Trans R Soc Trop Med Hyg.

[55] Wongsrichanalai C, Arevalo I, Laoboonchai A, Yingyuen K, Miller RS (2003). Rapid diagnostic devices for malaria: field evaluation of a new prototype immunochromatographic assay for the detection of *Plasmodium falciparum* and non-*Plasmodium falciparum*.. Am J Trop Med Hyg.

[56] Worth CB, De Meillon B (1960). Culicine mosquitoes (Diptera: *Culicidae*) recorded from the province of Mocambique (Portuguese East Africa) and their relationship to arthropod-borne viruses.. Anais Inst Med Trop.

[57] Linthicum KJ, Davies FG, Kairo A (1985). Rift Valley Fever virus (family Bunyaviridae, genus *Phlebovirus*) Isolation from Diptera collected during an inter-epizootic period in Kenya.. J Hyg Camb.

